# The Hsp70/Hsp90 Chaperone Machinery in Neurodegenerative Diseases

**DOI:** 10.3389/fnins.2017.00254

**Published:** 2017-05-16

**Authors:** Rachel E. Lackie, Andrzej Maciejewski, Valeriy G. Ostapchenko, Jose Marques-Lopes, Wing-Yiu Choy, Martin L. Duennwald, Vania F. Prado, Marco A. M. Prado

**Affiliations:** ^1^Molecular Medicine, Robarts Research Institute, University of Western OntarioLondon, ON, Canada; ^2^Program in Neuroscience, University of Western OntarioLondon, ON, Canada; ^3^Department of Biochemistry, University of Western OntarioLondon, ON, Canada; ^4^Department of Pathology and Laboratory Medicine, University of Western OntarioLondon, ON, Canada; ^5^Department of Physiology and Pharmacology, University of Western OntarioLondon, ON, Canada; ^6^Department of Anatomy and Cell Biology, Schulich School of Medicine and Dentistry, University of Western OntarioLondon, ON, Canada

**Keywords:** STIP1, HOP, Alzheimer's disease, tau, ALS, Parkinson's disease, Huntington's disease, TDP-43

## Abstract

The accumulation of misfolded proteins in the human brain is one of the critical features of many neurodegenerative diseases, including Alzheimer's disease (AD). Assembles of beta-amyloid (Aβ) peptide—either soluble (oligomers) or insoluble (plaques) and of tau protein, which form neurofibrillary tangles, are the major hallmarks of AD. Chaperones and co-chaperones regulate protein folding and client maturation, but they also target misfolded or aggregated proteins for refolding or for degradation, mostly by the proteasome. They form an important line of defense against misfolded proteins and are part of the cellular quality control system. The heat shock protein (Hsp) family, particularly Hsp70 and Hsp90, plays a major part in this process and it is well-known to regulate protein misfolding in a variety of diseases, including tau levels and toxicity in AD. However, the role of Hsp90 in regulating protein misfolding is not yet fully understood. For example, knockdown of Hsp90 and its co-chaperones in a *Caenorhabditis elegans* model of Aβ misfolding leads to increased toxicity. On the other hand, the use of Hsp90 inhibitors in AD mouse models reduces Aβ toxicity, and normalizes synaptic function. Stress-inducible phosphoprotein 1 (STI1), an intracellular co-chaperone, mediates the transfer of clients from Hsp70 to Hsp90. Importantly, STI1 has been shown to regulate aggregation of amyloid-like proteins in yeast. In addition to its intracellular function, STI1 can be secreted by diverse cell types, including astrocytes and microglia and function as a neurotrophic ligand by triggering signaling via the cellular prion protein (PrP^C^). Extracellular STI1 can prevent Aβ toxic signaling by (i) interfering with Aβ binding to PrP^C^ and (ii) triggering pro-survival signaling cascades. Interestingly, decreased levels of STI1 in *C. elegans* can also increase toxicity in an amyloid model. In this review, we will discuss the role of intracellular and extracellular STI1 and the Hsp70/Hsp90 chaperone network in mechanisms underlying protein misfolding in neurodegenerative diseases, with particular focus on AD.

## Brief introduction to chaperones and co-chaperones

A major requirement for cellular growth, function, and survival is the proper folding, maturation, and degradation of proteins. These activities are carried out by molecular chaperones, many of which are heat shock proteins (Hsps). The heat shock response was first discovered in the early 1960s in *Drosophila* that displayed changes in salivary gland transcriptional activity in response to different incubation temperatures (Ritossa, 1962). It was not until 1974 that Hsps were discovered and interest in this field of biology became widespread (Tissieres et al., 1974). Transcription of heat shock genes is mostly regulated by heat shock factor 1 (HSF1). Inactive HSF1 is localized in the cytosol, but upon heat stress translocates to the nucleus and binds to promoters of heat shock elements, inducing transcription and leading to an increase in Hsp expression (Morimoto, 1998). Activation of HSF1 and subsequent shuttling to the nucleus is a typical stress response and also allows for control of cell cycle, protein translation and glucose metabolism (Dai et al., 2007). It is now well-accepted that Hsps not only aid in mediating cellular responses to stress, but are also critical in general protein quality control. Some of the major roles of molecular chaperones include the regulation of the unfolding protein response due to stress, degradation of misfolded or aggregated proteins, regulation of macromolecular complexes, and protein-protein interactions.

There are several major classes of Hsps involved in the protein quality control machinery: Hsp60, Hsp70 and Hsp90, Hsp40, Hsp100, Hsp110, as well as the ATP-independent small heat shock proteins (sHsps) such as Hsp20, αA-crystallin, and αB-crystallin. Hsp40, also known as DnaJ, is commonly found acting as a co-chaperone for Hsp70 and regulates ATP-dependent polypeptide binding to Hsp70, prevention of premature polypeptide folding, and ATPase activity of Hsp70 (Cyr et al., 1992; Frydman et al., 1994; Tsai and Douglas, 1996). In yeast, the family of Hsp100 proteins protect cells from extreme physiological and environmental stress (Sanchez et al., 1992; Glover and Lindquist, 1998) and have the unique ability to re-solubilize aggregated insoluble proteins (Parsell et al., 1994). In metazoans disaggregase activity is carried out by the tricomplex of Hsp70, a J Protein and Hsp110 (Shorter, 2011; Rampelt et al., 2012; Gao et al., 2015). For the purpose of this review, we will focus mainly on the roles of Hsp70 and Hsp90 as well as of the critical co-chaperone stress-inducible phosphoprotein I (STI1, STIP1) and their regulation of protein misfolding and signaling in neurodegenerative diseases. Comprehensive discussion of different chaperones including their roles in the ER can be found in excellent recent reviews elsewhere (McLaughlin and Vandenbroeck, 2011; Marzec et al., 2012; Melnyk et al., 2015; Ellgaard et al., 2016).

Hsp70 and Hsp90 and homologs are both widely expressed in some lower order prokaryotes and in all eukaryotes, with Hsp90 constituting ~1% of all cellular proteins in eukaryotes (Borkovich et al., 1989). Hsp90 activity is regulated through interactions with a large network of co-chaperones providing quality control of a wide range of client proteins. Initially, client proteins are recruited by Hsp40 and Hsp70 and then transferred to Hsp90 by the co-chaperone STI1 (the human homolog is also known as Hsp-organizing protein or HOP; Lassle et al., 1997; Chen and Smith, 1998; Johnson et al., 1998; Taipale et al., 2010). Recent studies suggest that Hsp90 has an important role in neurodegeneration. Pharmacological inhibition of Hsp90 results in Hsp70 and Hsp40 upregulation, which can control the expression of several synaptic proteins, but it can also channel misfolded protein for degradation by the proteasome (Luo et al., 2007; Chen et al., 2014; Wang et al., 2016). Protein aggregation is a major hallmark of several neurodegenerative diseases, including Alzheimer's disease (AD), Amyotrophic lateral sclerosis (ALS), Parkinson's disease (PD), Huntington's disease (HD), and Creutzfeldt-Jakob disease (CJD). Therefore, the chaperone machinery is becoming a major therapeutic target across these diseases.

## Hsp70

The Hsp70 family of proteins is a class of highly abundant and ubiquitously expressed chaperones that participate in many biological processes, including protein trafficking, early stages of nascent polypeptide folding and the refolding or degradation of aggregated peptide products (Bukau et al., 2006). There are several eukaryotic Hsp70 isoforms (reviewed in Kabani and Martineau, 2008). Hsp70 is composed of two distinct domains, a 40 kDa N-terminal nucleotide-binding domain (NBD) that regulates client association and a 25 kDa C-terminal substrate-binding domain (SBD), which recognizes exposed hydrophobic stretches in the early stages of client protein folding (Rudiger et al., 1997; Bukau et al., 2006). A short, flexible hydrophobic linker joins both domains (Jiang et al., 2005). ATP binding and hydrolysis is coupled to allosteric changes in Hsp70, which influence protein-client interactions in the Hsp70 chaperone cycle (Mayer et al., 2000). Post-translational modification of Hsp70 by phosphorylation at T504 and/or acetylation at several serine residues promotes dimerization of Hsp70 (Morgner et al., 2015). Furthermore, presence of a client and Hsp40 supports dimerization of Hsp70 and substrate binding (Morgner et al., 2015).

ATP binding to the NBD results in the opening of a SBD α-helical lid, stimulating binding of substrate proteins through interactions with the NBD and SBD (Jiang et al., 2005). The ADP-bound state results in the closing of the α-helical lid over the substrate-binding cleft and stabilizes client association (Schlecht et al., 2011). The Hsp70 chaperone cycle is inherently slow due to the low ATPase activity of Hsp70 (Swain et al., 2007). Thus, a family of J proteins recruits the client to Hsp70 and stimulates the Hsp70 ATPase activity (Misselwitz et al., 1998). The J protein then dissociates from the ternary complex and a nucleotide-exchange factor releases the bound ADP from Hsp70 returning it to the apo-conformation. This leaves the NBD available for recruitment of ATP, upon which the α-helical lid can “open” and release the client peptide (Misselwitz et al., 1998; Schlecht et al., 2011). The cycle repeats in an interactive process until the client peptide adopts its native structure or is passed on to another part of the chaperone machinery.

Hsp70 class of chaperones typically recognizes client proteins in the early stages of folding through short hydrophobic sequences rich in leucine residues (Rudiger et al., 1997). Such hydrophobic stretches are greatly exposed during the early stages of protein translation and folding, leading to unfavorable intra- and inter-molecular interactions (Hartl and Hayer-Hartl, 2009). Hsp70 binding to client proteins in the early stages of protein folding controls the availability of such regions, facilitating formation of the proper protein fold, while inhibiting aggregate formation. If proper folding of the client is not possible, Hsp70 association with additional co-chaperones promotes degradation of the misfolded protein (Meacham et al., 2001; Petrucelli et al., 2004; Jana et al., 2005; Dickey et al., 2007; Muller et al., 2008). The abundance of exposed hydrophobic stretches in proteins prone to self-association in neurodegenerative diseases draws parallel to proteins in early stages of folding, which indicates a potential role of the Hsp70/Hsp90 machinery in modulating pathogenic aggregate formation (Muchowski and Wacker, 2005; Hartl and Hayer-Hartl, 2009).

## Hsp90

**Figure 1:**
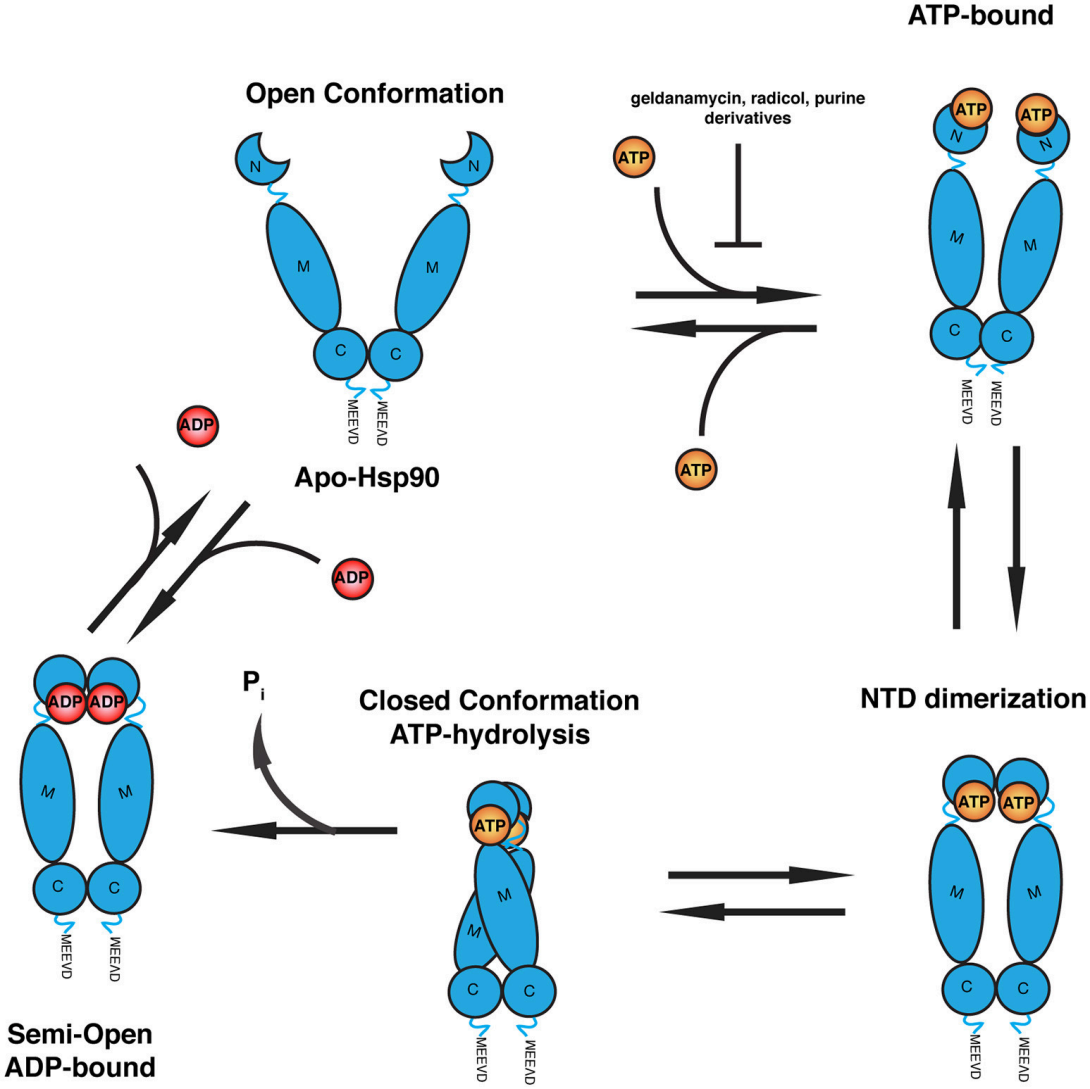
**Schematic of the ATPase cycle of Hsp90**. Hsp90 homodimer initially adopts an open V-shaped conformation. Binding of ATP to the N-terminal ATPase domain induces a conformational change where the N-terminal lids close and ATP is cradled in the nucleotide-binding pocket. This induces dimerization of the N-terminal domains of each homodimer followed by closure of Hsp90 and recruitment of the M domain for ATP hydrolysis. The dimers dissociate into a semi-open intermediate state with ADP bound. Release of ADP dissociates the N-termini to allow repetition of the ATPase cycle. Common Hsp90 inhibitors (geldanamycin, radicicol, and purine derivatives) bind to the N-terminus of Hsp90 and compete with ATP for binding.

Hsp90 is a highly conserved molecular chaperone from yeast to mammals and it is essential for proper folding and maintenance of its client proteins in eukaryotic cells (see Figure 1). Over 500 physical and genetic interactions have been identified in yeast spanning diverse families of cellular proteins, which include transcription factors, steroid hormone receptors, and protein kinases (Zhao et al., 2005; McClellan et al., 2007). Through concerted efforts involving other chaperones and co-chaperones it drives the final maturation of client proteins. There are two major isoforms of Hsp90 identified in the cytosol, inducible Hsp90α and constitutively expressed Hsp90β, as well as Grp94 in the endoplasmic reticulum (Csermely et al., 1998).

Hsp90 forms a homodimeric biologically functional unit composed of three distinct regions connected by flexible linker. Each protomer contains a highly-conserved N-terminal domain (NTD) responsible for nucleotide binding, a middle domain (MD) important for client recognition and ATP hydrolysis and a C-terminal domain (CTD), which is the primary site responsible for dimerization (Pearl and Prodromou, 2006; Taipale et al., 2010). Additionally, the CTD contains a conserved “MEEVD” sequence used for interactions with the large tetratricopeptide repeat domain class of co-chaperone proteins, which regulate Hsp90 protein activity (Young et al., 1998).

The NTD contains a deep ATP/ADP binding pocket evolutionarily conserved amongst the GHKL (gyrase, Hsp90, histidine kinase, MutL) ATPase superfamily (Meyer et al., 2003). The binding pocket is composed of multiple α-helices and a β-sheet flanking the bound ATP molecule (Prodromou et al., 1997). Intramolecular recruitment of the MD on each protomer brings critical residues into contact forming the required split ATPase active site necessary for ATP hydrolysis (Ali et al., 2006). The N-terminal region contains an additional molecular lid mechanism, which crosses over the nucleotide-binding pocket in the ATP-bound state and holds the nucleotide in place, but remains in the open conformation upon ADP-binding (Ali et al., 2006). See Figure 1 for an overview of the Hsp90 conformations when bound to ADP or ATP.

Nucleotide binding and hydrolysis are directly coupled to large structural rearrangements in Hsp90 that are regulated through interactions with client and co-chaperone proteins (Csermely et al., 1993; Kirschke et al., 2014; Lavery et al., 2014). Apo-Hsp90 presents an “open” conformation where the two protomers form a V-shaped dimer (Vaughan et al., 2010). Nucleotide binding induces an intermediate-state where the N-terminal lid cradles ATP within the nucleotide-binding pocket. This results in a second repositioning of the N-terminal region favoring its dimerization and recruitment of the MD for ATP hydrolysis through a conserved arginine (R380 in yeast) that contacts the γ-phosphate of ATP (Cunningham et al., 2012). Finally, ATP is hydrolyzed and Hsp90 reverts to the “open” conformation where the N-terminal domains dissociate, allowing for the repetition of the cycle. ATP hydrolysis and the structural rearrangements in Hsp90 during client refolding are influenced and regulated by a diverse set of co-chaperones (Zuehlke and Johnson, 2010). These co-chaperones have varying effects on the ATPase activity and conformational rearrangements in Hsp90. Post-translational modifications of Hsp90 include acetylation, nitrosylation, phosphorylation, and methylation, which has been elegantly reviewed by Li and Buchner (2013).

While ATPase induced conformational rearrangements in Hsp90 are well-understood, their influence on client protein folding remains enigmatic. Hsp90-directed folding occurs late during the folding of nascent peptides. Structural studies involving binding to intrinsically disordered tau suggest that substrate recognition occurs through low-affinity hydrophobic interactions between the client protein and a large substrate-binding interface on Hsp90 (Zuehlke and Johnson, 2010; Karagoz et al., 2014). This mechanism allows for detection of scattered exposed hydrophobic patches in protein folding intermediates. Interestingly, Hsp90 affinity for tau appears to be independent of ATP binding as both the apo and ATP-bound forms possess equal affinities for tau (Karagoz et al., 2014).

The prominent role and poor prognosis of Hsp90 overexpression in various cancers has led to the development of a number of Hsp90 inhibitors (Roe et al., 1999; Trepel et al., 2010). Hsp90 inhibition results in the downregulation of many oncogenic client proteins that require Hsp90 for maturation. The most prominent drugs inhibit the ATPase activity of Hsp90 and are based on the geldanamycin, radicicol, or purine derivatives, which function as competitive inhibitors of ATP binding to Hsp90 (Roe et al., 1999; Sidera and Patsavoudi, 2014). Initial geldanamycin and radicicol derivatives proved to be potent inhibitors of Hsp90; however, their therapeutic value is low due to severe toxicity by targeting Hsp90 in normal cells (Jhaveri et al., 2012; Trendowski, 2015).

Co-chaperones along with individual Hsps comprise a chaperone network that is altered in malignancy and neurodegeneration (Workman et al., 2007; Moulick et al., 2011; Lindberg et al., 2015; Rodina et al., 2016). Targeting specific co-chaperones or chaperone complexes may help to avoid cytotoxicity by directly targeting Hsp90 activity (Yi and Regan, 2008; Moulick et al., 2011; Rodina et al., 2016). The purine derivative PU-H71 possesses unique selectivity amongst Hsp90 inhibitors, preferentially targeting high-molecular-weight complexes composed of Hsp70/90 and various co-chaperones and client proteins, which are enriched in numerous malignant cell models, but absent in non-oncogenic tissue (Moulick et al., 2011; Rodina et al., 2016). The formation of these large stable chaperone species appears to be cancer specific and diagnostic proteomic approaches may serve as a method to clinically screen patients that are most likely to benefit from targeting such species. Whether large and stable chaperone complexes with misfolded proteins occur in different neurodegenerative diseases is currently unclear. Indeed, therapeutic approaches targeting chaperones in neurodegeneration still fall behind from those in cancer cells.

Hsp70 and Hsp90 both interact with many co-chaperones containing tetratricopeptide repeat (TPR) domains, which consist of three or more 34-amino acid residues (Lamb et al., 1995). These motifs form anti-parallel α-helices (Allan and Ratajczak, 2011) that bind to the C-terminus of the chaperone and are the main interaction site for co-chaperones (Smith, 2004), along the EEVD peptide motif on Hsp70 and Hsp90 (Kajander et al., 2009). Proteins containing TPR domains typically share no other sequence homology, but are commonly found to be involved in regulation of cell cycle, protein trafficking, phosphate turnover, and transcriptional events (Blatch and Lassle, 1999). TPR domain-containing co-chaperones regulate the ATP cycle of chaperones and aid in client transport to binding pockets, where they are folded. Hsp40 may help coordinate other co-chaperones in binding Hsp70, such as Hsp70-interacting protein (Hip; Hohfeld et al., 1995) in the early stages of the chaperone cycle, as well as STI1 and SGT (Allan and Ratajczak, 2011). STI1 is also a co-chaperone for Hsp90, along with p23, Cdc37, and the immunophilins peptidyl-prolyl cis-trans (PPIases) isomerases FKBP51 and FKBP52, phosphatase PP5 and the cyclophillin Cyp40 (Allan and Ratajczak, 2011). Some of these co-chaperones inhibit Hsp90 ATP turnover (Rehn and Buchner, 2015). C-terminal Hsp70-interacting protein (CHIP) is also a co-chaperone for both Hsp70 and Hsp90. In this review, we will focus mainly on the roles of Hsp70, Hsp90, and the co-chaperone STI1 in protein misfolding. We will also discuss the unique cytokine-like activities of STI1. Importantly, both the extracellular and intracellular activities of STI1 seem to converge to increase cellular resilience (Beraldo et al., 2013). Moreover, we will briefly describe some of the co-chaperones that may also have a role in protein misfolding diseases, such as CHIP and high molecular weight immunophilins.

## Hsp70/Hsp90 partners in neurodegenerative diseases

There is a number of Hsp70 and Hsp90 co-chaperones that have implications for neurodegenerative diseases. High molecular weight FK506-binding proteins (FKBPs) FKBP51 and FKBP52 impose a variety of effects on tau structure and function, which will be discussed further in the AD subsection of this review. Another co-chaperone, a high molecular weight PPIase Cyp40, can bind Aβ and regulate import of Aβ into mitochondria. Interestingly, inhibiting Cyp40 was found to be protective against Aβ-toxicity in mitochondria and neurons in an amyloid-precursor protein (APP) transgenic mouse model (Du and Yan, 2010). This suggests that Aβ may be regulated by an Hsp90/PPIase complex, but further investigation is required. For a more extensive review on PPIases in regulating the levels and toxicity of proteins in AD, see excellent review by Blair et al. (2015).

The Hsp90 co-chaperone p23 typically comes into play in a mature Hsp90-client complex (Felts and Toft, 2003), whereby it inhibits ATP turnover on Hsp90 (Rehn and Buchner, 2015). In the context of AD, p23 has been found to bind γ-secretases and to promote the non-amyloidogenic pathway of amyloid precursor protein (ultimately reducing production of Aβ species; Vetrivel et al., 2008) and silencing of p23 gene expression reduced the levels of total tau and phosphorylated tau (Dickey et al., 2007).

**Figure 2:**
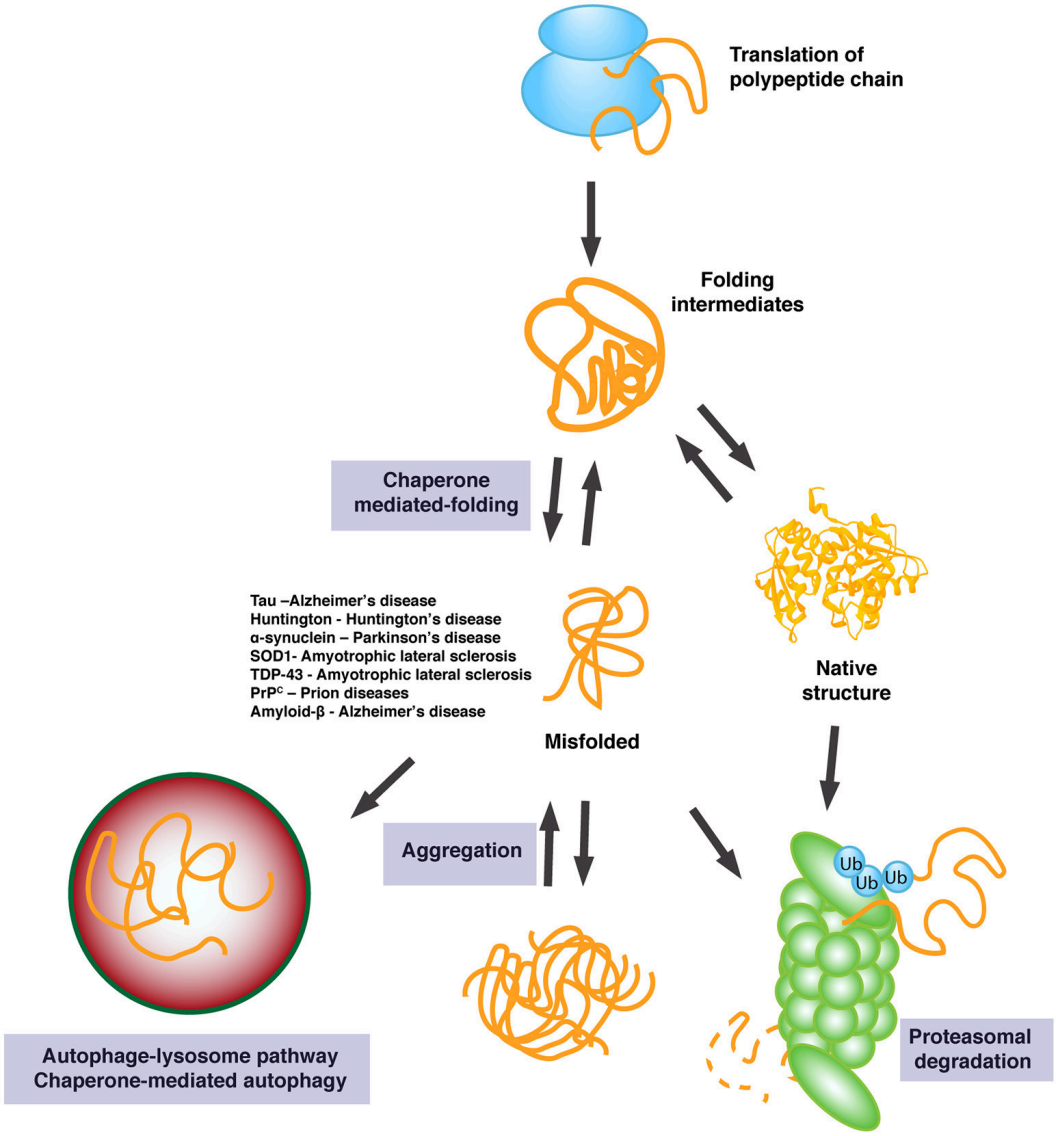
**Outline of the chaperone response in protein folding**. Chaperones facilitate proper folding of a diverse array of client proteins and prevent oligomer and aggregate formation. If folding is not possible, misfolded proteins are targeted for protein degradation to maintain proper protein homeostasis. Degradation is achieved through the ubiquitin-proteasome system (UPS) or the autophagy-lysosome pathway. A list of diseases and the associated aggregate discussed in this review are outlined.

Hsp70 and Hsp90 are critical to folding and maturation of a number of clients, but they are also key players in regulation of the proteasome, a simplified representation of this activity can be found in Figure 2. CHIP, a Hsp70-Hsp90 co-chaperone, is an E3 ubiquitin ligase (Ballinger et al., 1999; McDonough and Patterson, 2003) that promotes client degradation via the proteasome. CHIP contains three TPR motifs at its N-terminus, a middle domain of charged residues (Ballinger et al., 1999) and a C-terminus containing a U-Box domain (Zhang et al., 2005). U-Box domains are characteristic of proteins involved in ubiquitination (Aravind and Koonin, 2000). Within this pathway, ubiquitin molecules are covalently attached to the protein of interest, and then recruited to the proteasome complex for degradation. CHIP interacts with Hsp70 and Hsp90 via TPR domain and then can bind with the substrate protein of interest, promoting substrate ubiquitination. Of note, CHIP is capable of binding tau and is responsible for its ubiquitination (Petrucelli et al., 2004), a critical activity that may be of importance for tauopathies.

Transcriptional regulation of the heat shock response is mediated by HSF1. HSF1 activity is dependent upon levels of chaperones and misfolded proteins, and other environmental stressors such as heat, aging, and changes in osmosis. Alternatively, Hsp70 and Hsp90 can negatively regulate the activation of HSF1, suppressing the heat shock response, allowing for a recovery period after the stressor is no longer present. Once in the nucleus, HSF1 trimerizes and binds Hsp gene promoters to activate transcription. Dai et al. (2003) further showed that this trimerization of HSF1 is mediated by CHIP, since both localize to the nucleus in response to cellular stress and form a complex once HSF1 is bound to DNA. Moreover, once degradation of misfolded proteins is complete, CHIP begins to degrade Hsp70 (unbound to any client). Therefore, CHIP stimulates HSF1 upregulation of Hsp70 in stress conditions and mediates its degradation to basal levels in the recovery period from stress.

### STI1/HOP co-chaperone structure and function

**Figure 3:**
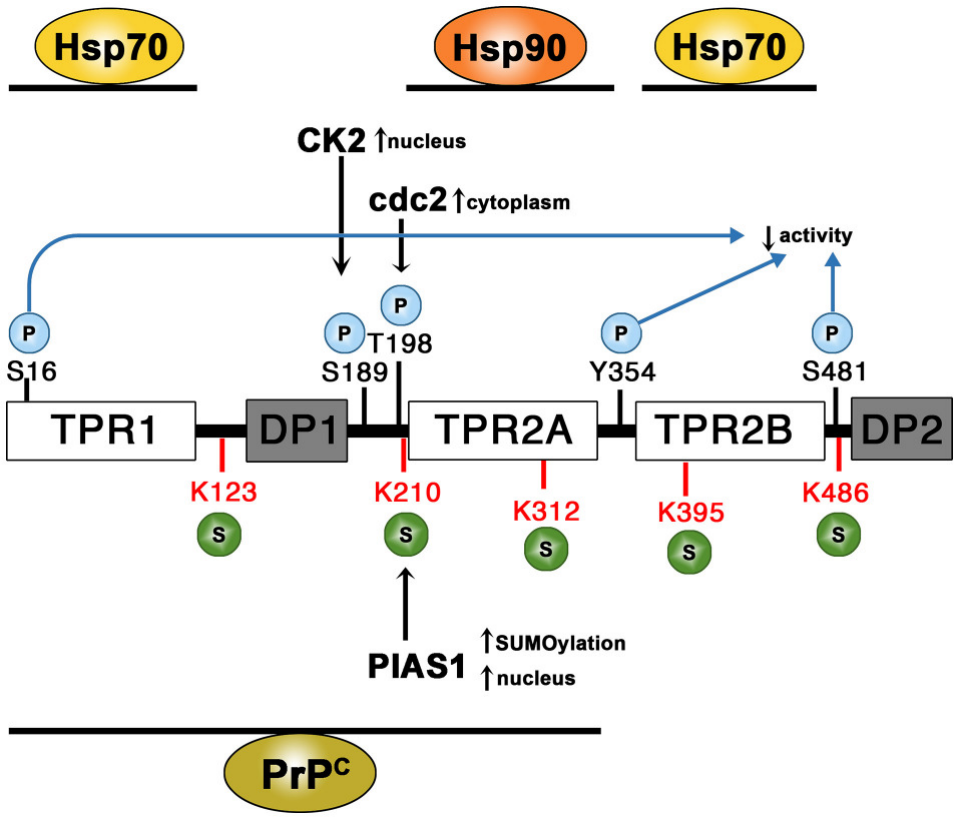
**Domain structure of STI1 and sites of post-translational modifications (PTM)**. STI1 is composed of three structurally similar tetratricopeptide repeat domains (TPR1, TPR2A, and TPR2B) and two regions rich in aspartate and proline residues (DP1 and DP2). The protein is subject to phosphorylation (S16, S189, T198, Y354, and S481). CK2 phosphorylation at S189 induces STI1 accumulation in the nucleus. In contrast phosphorylation by cdc2 at T198 localizes STI1 to the cytoplasm. Five possible SUMOylation sites have been identified (K123, K210, K312, K395, and K486). SUMOylation by PIAS1 at K210 may stimulate SUMOylation at the alternate sites. Association of PIAS1 with STI1 by a SUMO-independent mechanism increases STI1 nuclear accumulation. Regions that bind HSP70, HSP90, and PrP^C^ are illustrated.

STI1 was discovered in 1989, as a protein that is upregulated during cellular stress in yeast (Nicolet and Craig, 1989). STI1 is a modular protein composed of three TPR domains (TPR1, TPR2A and TPR2B) and two domains rich in aspartate and proline residues (DP1 and DP2, see Figure 3). The TPR domains of STI1 bind Hsp70 and Hsp90 to facilitate client protein transfer. While the C-terminal domain of Hsp90 is critical for the binding of TPR domains, additional contacts are made with the middle domain of Hsp90 (Lee et al., 2012; Schmid et al., 2012). Binding of Hsp90 by STI1 results in non-competitive inhibition of its ATPase activity through interaction with TPR2A-TPR2B fragment and stabilizes Hsp90 in an open conformation. However, the human homolog of STI1, HOP, appears to be ~10-fold less potent as an inhibitor of the Hsp90 ATPase activity (Prodromou et al., 1999; Richter et al., 2008). Hsp70 and Hsp90 engagement is facilitated through sequential interactions with the individual TPR domains of STI1 (Rohl et al., 2015b). The function of the DP domains (DP1 and DP2) is less clear. The minimal fragment of STI1 that supports client activation is composed of TPR2A-TPR2B-DP2 (Schmid et al., 2012; Rohl et al., 2015b). The *Caenorhabditis elegans* STI1 homolog, CeHOP, contains only the TPR2A-TPR2B-DP2 module, but is still capable of binding Hsp70 and Hsp90, though not simultaneously (Gaiser et al., 2009). Deletion of CeHOP is not lethal, but mutant worms have deficits in sexual development and reduced resistance to heat stress (Gaiser et al., 2009; Song et al., 2009). Functionality of CeHOP suggests the TPR1-DP1 module may be dispensable for client activation *in vivo* (Gaiser et al., 2009).

Interestingly, STI1-based constructs lacking the DP2 domain did not support glucocorticoid receptor activation, one of STI1 functions in yeast cells (Schmid et al., 2012). X-ray crystallographic structures of the TPR2A-TPR2B domain revealed the linker between these domains is quite rigid. As a result, the TPR domains adopt an S-shaped form with their hydrophobic clefts responsible for binding the C-terminal Hsp90 residues oriented in opposite directions (Schmid et al., 2012). Complementary NMR spectroscopy experiments revealed that additional inter-domain contacts are formed between the C-terminal helix of TPR2B, the linker connecting TPR2B to the DP2 domain and α-helices 1 and 2 of the DP2 domain (Rohl et al., 2015b). These additional contacts form a rigid C-terminus composed of TPR2A-TPR2B-DP2. These results indicate that DP2 contributes to the quaternary structure of STI1, underlying its importance in STI1 function.

STI1 possesses two Hsp70 binding sites located in TPR1 and TPR2B; however, it binds to Hsp70 in a 1:1 stoichiometry (Scheufler et al., 2000; Rohl et al., 2015b). The current model for STI1 function in Hsp70 and Hsp90 coordination proposes that in the absence of Hsp90, the TPR2B domain represents the high-affinity binding site for Hsp70, these interaction sites are shown in Figure 3. Hsp90 binding induces a more “open” conformation between TPR1-DP1 fragment and the functional C-terminal TPR2A-TPR2B-DP2 domains. Hsp90 binding to TPR2A reduces accessibility of Hsp70 binding to TPR2B, thus the TPR1 domain becomes the predominant binding site for Hsp70 in the ternary complex (Rohl et al., 2015b). Binding of Hsp90 may also reorient the TPR1-DP1 module into close proximity to TPR2B, presumably facilitating transfer of client protein (Rohl et al., 2015b). Thus, the length of the linker bridging TPR1-DP1 to TPR2A-TPR2B-DP2 impacts STI1 function in client refolding (Rohl et al., 2015b). Deletion of the linker results in decreased formation of ternary complexes of STI1-Hsp70-Hsp90 and decreased protein client activation *in vivo* (Rohl et al., 2015b).

STI1 is widely expressed in most tissues and is typically localized in the cytoplasm, but can be found associated with the Golgi (Honore et al., 1992) and in the nucleus (Longshaw et al., 2004; Beraldo et al., 2013; Soares et al., 2013). Nuclear accumulation of STI1 is characteristic of stressed cells (Beraldo et al., 2013).

STI1 is subject to posttranslational modifications, which regulate its co-chaperone activity (Rohl et al., 2015a). Five different phosphorylation sites have been identified in the human STI1 homolog HOP corresponding to S16, S189, T198, Y354, and S481, see Figure 3 for simplified STI1 structure and respective phosphorylation sites. Phosphomimetic mutations resulted in decreased glucocorticoid receptor activation *in vivo* and Hsp70 binding affinities indicating that phosphorylation regulates STI1 co-chaperone function (Rohl et al., 2015a). Interestingly, Y354E phosphomimetic variant located in the loop joining TPR2A-TPR2B appeared to disrupt the rigid linker joining the two domains and promotes a more dynamic flexibility causing loss of function (Rohl et al., 2015a).

STI1 is typically found in the cytosol, but it can shuttle between the nucleus and cytoplasm (Longshaw et al., 2004), due to the presence of a nuclear localization signal (NLS) at amino acids 222–239 (of mouse STI1). Specifically, phosphorylation of STI1 by casein kinase II (CKII) and cell division cycle kinase II (cdc2) at S189 and T198 (respectively), contiguous to STI1 NLS (Longshaw et al., 2000) regulates nuclear localization of STI1 (Longshaw et al., 2004). CKII stimulates cellular growth by promoting cells to enter G1 phase of cell cycle (Pyerin, 1994), whereas cdc2 favors cell division (Matsumoto and Fujimoto, 1990). The rate of STI1 export from the nucleus is much higher than import, which can be inhibited with leptomycin B, a nuclear export inhibitor (Longshaw et al., 2004). Of particular interest, STI1 interacts with the nuclear small ubiquitin-like modifier (SUMO) E3 ligase family protein inhibitor of activated STAT (PIAS) and many other components of the SUMOylation machinery (Soares et al., 2013), suggesting potential STI1 regulation of genotoxic stress responses. STI1 was retained in the nucleus of astrocytes overexpressing PIAS1 (Soares et al., 2013) and Hsp90 was also found to be appreciably colocalized with STI1 and PIAS1 in the nucleus. Specifically, PIAS1 can poly-SUMOylate STI1 at several lysine residues: K123, K210, K312, K395, and K486 (depicted in Figure 3; Soares et al., 2013) and it is proposed that K210 hierarchically regulates SUMOylation of the other sites. Co-transfection of HEK293 cells with SUMO, PIAS1, and STI1 increased SUMOylation of STI1, but it was the interaction between PIAS1 and STI1 that supported nuclear retention, not SUMOylation. This suggests that nuclear localization of STI1 may alter its co-chaperone activities with Hsp90 and can help with nuclear protein crowding in particular sites that facilitate protein-protein interactions, such as PML nuclear bodies (Soares et al., 2013). However, STI1 has also been shown to function as a scaffold to recruit Hsp90 for other nuclear functions, including regulation of canalization or developmental robustness, by controlling Piwi and regulating Piwi-interacting RNA and impacting transposons (Gangaraju et al., 2011; Karam et al., 2017).

Deletion of STI1 is not lethal in yeast (Flom et al., 2007) and in *C. elegans* elimination of STI1 caused reduced lifespan (Song et al., 2009). Interestingly, knockout of STI1 in mice is embryonically lethal by E10.5 (Beraldo et al., 2013). Half of STI1-null blastocysts also failed to thrive, suggesting a key role for STI1 early in development (Beraldo et al., 2013). Homozygous embryos have a 50% reduction in Hsp90 client protein expression (p53, GRK2, STAT3—all clients that when knocked out are embryonically lethal; Beraldo et al., 2013). Mouse embryonic fibroblast lacking STI1 also failed to thrive in culture. Moreover, astrocytes derived from STI1 haplo-sufficient mice are also less resilient to irradiation (Soares et al., 2013), and neurons are less resistant to oxygen-glucose deprivation (Beraldo et al., 2013) or β-amyloid toxicity (Ostapchenko et al., 2013). This indicates that in higher organisms, STI1 is essential for regulating cellular resilience.

### Cytokine-like activity of STI1

**Figure 4:**
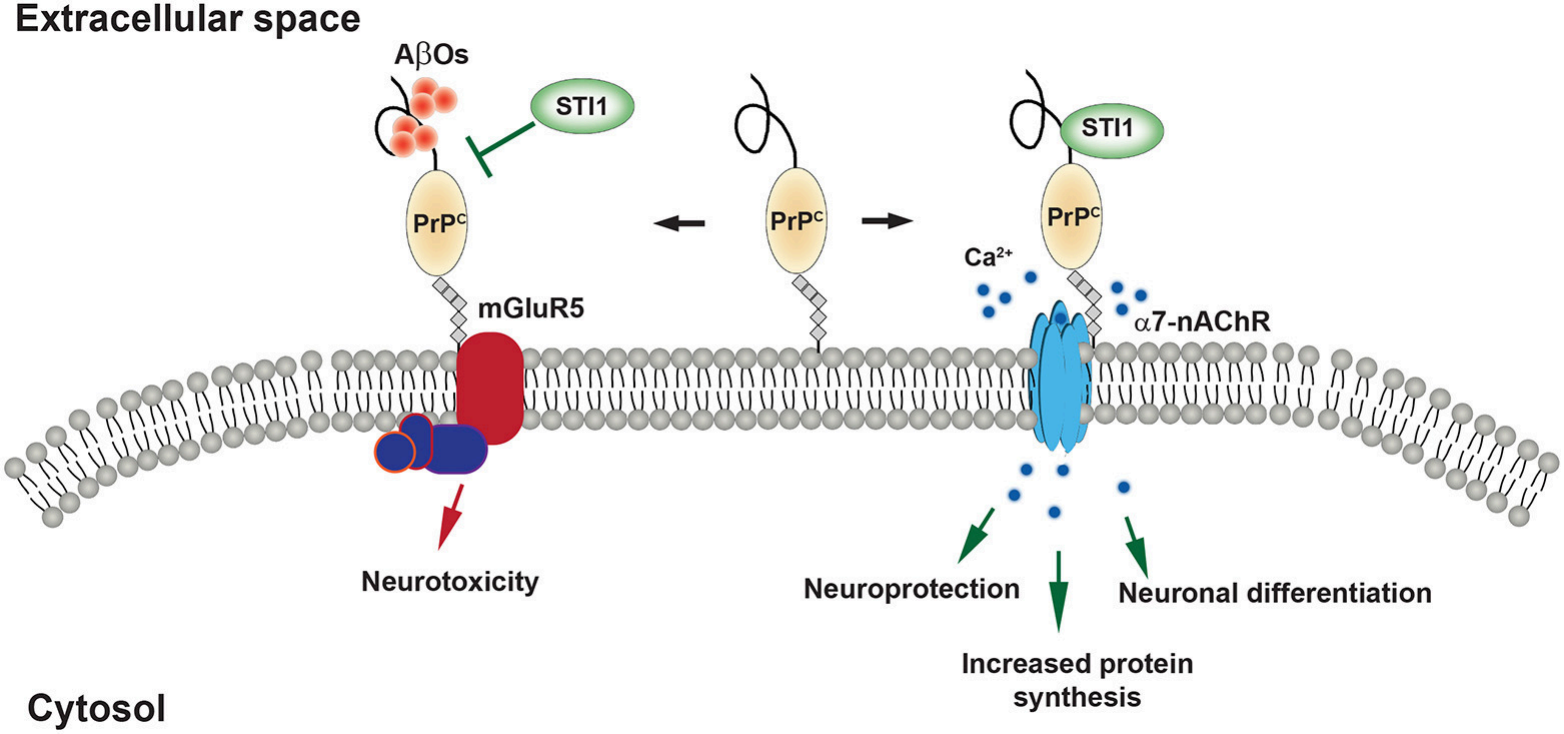
**STI1 signaling mediated by the cellular prion protein (PrP^C^). (Right)** PrP^C^ binding to extracellular STI1 induces neuroprotective and neuro- differentiation through Ca^2+^ influx via α7-nAChR. **(Left)** Aβ oligomers transmit toxic signaling events through PrP^C^. STI1 inhibits Aβ oligomer binding to PrP^C^ and/or activate protective signaling events.

STI1 is a critical co-chaperone in the functionality of the Hsp70/Hsp90 machinery in cells. However, there is also a large body of literature that focuses on the extracellular effects of STI1, specifically the consequences of interaction with the cellular prion protein (PrP^C^; Zanata et al., 2002; Lopes et al., 2005; Caetano et al., 2008; Arantes et al., 2009; Beraldo et al., 2010, 2013; Roffe et al., 2010; Hajj et al., 2013; Ostapchenko et al., 2013; Maciejewski et al., 2016), which is illustrated in Figure 4. PrP^C^ is anchored to cell membranes by a glycosylphosphatidylinositol (GPI) moiety and it is thought to serve as a molecular scaffolding protein organizing signaling complexes (Linden et al., 2008).

STI1 must be secreted in order to interact with PrP^C^ at the cell surface. Like Hsp70 and Hsp90 (Clayton et al., 2005; Lancaster and Febbraio, 2005), STI1 can be secreted via exosomes (Hajj et al., 2013). PrP^C^ is also secreted by exosomes and both PrP^C^ and STI1 can be found on the surface of the exosomes derived from astrocytes (Hajj et al., 2013). Secreted STI1 can then interact with and bind to the surface of neurons (Hajj et al., 2013), having a variety of effects on cell growth and survival, in a PrP^C^-dependent manner.

Martins, Linden and Brentani (Chiarini et al., 2002; Zanata et al., 2002) were the first to report the interaction between STI1 and PrP^C^ and to describe STI1 as a signaling molecule. Notably, interaction between STI1 and PrP^C^ reduced apoptotic cell death induced by the protein synthesis inhibitor anisomycin. These effects were confirmed to be PrP^C^-dependent using PrP-null neurons or exogenous treatment with a truncated form of STI1 lacking a critical PrP^C^ binding site (STI1Δ230–245). Lopes et al. (2005) found that cultured neurons treated with exogenous STI1 are more resilient to protein synthesis inhibitors, but this increased neuronal resilience required activation of cAMP-PKA pathway. STI1-PrP^C^ engagement is also capable of stimulating neuronal differentiation and this was dependent upon activation of the mitogen-activated protein kinase (MAPK/ERK) signaling pathway (Lopes et al., 2005). Roffe et al. (2010) showed that STI1-PrP^C^ interaction increased protein synthesis in hippocampal neurons via mTOR and this was dependent upon the MAPK/ERK and PI3K signaling pathways. STI1 and PrP^C^ interact briefly at the cell surface and are quickly internalized by distinct cellular pathways, limiting the levels of signaling activation by STI1 (Caetano et al., 2008). Activation of PKA and ERK signaling cascades by STI1-PrP^C^ interaction is in part due to calcium signaling (Beraldo et al., 2010). STI1-PrP^C^ signaling in hippocampal neurons requires α7 nicotinic acetylcholine receptors and inhibition of these receptors with α-bungarotoxin or use of knockout neurons eliminates STI1-PrP^C^ neuroprotection (Beraldo et al., 2010; Ostapchenko et al., 2013). Finally, STI1-PrP^C^ association supports proliferation and pluripotency of neural stem cells and promotes neurosphere formation (Santos et al., 2011). These findings altogether suggest that STI1 is critical for proper growth, development, and resilience to cellular stress.

Decreased STI1 levels significantly reduces cellular tolerance and cells display stress phenotypes, such as increased STI1 nuclear localization and nuclear labeling for γ-H2AX, a marker for double stranded DNA breaks, as seen in germ-line knockdown of STI1 in Drosophila (Karam et al., 2017) and in mouse embryonic fibroblasts (Beraldo et al., 2013). Astrocytes derived from STI1 haplo-insufficent mice also secrete 50% less STI1, which has consequences on PrP^C^-dependent resilience. Exogenous treatment with recombinant STI1 in neuronal cultures subjected to oxygen-glucose deprivation (OGD) reduced levels of cellular death in a PrP^C^ and α7 nicotinic receptor-dependent manner (Beraldo et al., 2013), further supporting the notion that extracellular STI1 is responsible for inducing some of these neuroprotective effects. Neuronal cultures from PrP-null animals were not protected from OGD upon STI1 treatment, further indicating that these effects are indeed PrP^C^-dependent.

Middle cerebral artery occlusion, a model for ischemic stroke was conducted on STI1-haplosufficient mice (Beraldo et al., 2013), which had increased mortality and a reliably larger infarct volume compared to control littermates. Additionally, Lee et al. (2013) found an upregulation of STI1 immunoreactivity in brains from MCAO rats and post-mortem tissue of stroke patients. STI1 contains a hypoxia response element within its promoter region, which can be activated by hypoxia-inducible factor 1-α (HIF1-α; Lee et al., 2013). This binding event is responsible for the elevated STI1 levels post-stroke, as knockout for HIF1-α or lentiviral shRNA administration to mice inhibited the increase in STI1 immunoreactivity around the infarct. Lee et al. (2013) also showed that STI1 increases proliferation of bone-marrow derived cells and recruits these cells to the areas of damage, as a means to promote and accelerate recovery. Together, these studies provide strong evidence that extracellular STI1 is required for recovery of post-ischemic insult and that exogenous treatment with STI1 could potentiate the recovery process.

It is educational also to learn how STI1 can signal in cancer cells, as some of the signaling pathways may be similar to those in neurons. In the context of cancer, increased proliferative capacity due to STI1 is a major problem. Wang et al. (2010) found that levels of STI1 were much higher in malignant vs. benign ovarian tumors. Additionally, serum levels of STI1 were ~6 times higher in ovarian cancer patients (Wang et al., 2010) and were being secreted by the cancerous cells. Activation of the ERK signaling pathway by secreted STI1 was responsible for the increased proliferative capacity of these cancerous cells. Elevated levels of STI1 were correlated with worsened prognosis in ovarian cancer patients (Chao et al., 2013), making this a useful biomarker for this type of cancer. Recently, Wang et al. (2017) found increased intracellular and extracellular levels of STI1 in renal cell carcinoma (RCCs) tumor cells. Increased proliferation was mediated by the activin A receptor, type II-like kinase 2 (ALK2), and the receptor regulated SMAD1/5 protein signaling cascade, independent of PrP^C^. Only differentiation of the osteoclasts from the RCCs was dependent upon STI1-PrP^C^ signaling. These studies provide further evidence that STI1 acts as a cytokine-like signaling molecule, promoting cellular growth by activating PrP^C^ dependent and independent pathways.

## Protein quality control in neurodegenerative diseases

Protein misfolding can lead to the formation of aggregates in diverse neurodegenerative diseases, such as AD, PD, ALS, frontotemporal dementia (FTD), HD, and prion diseases (Knowles et al., 2014). Protein aggregates are formed by highly ordered filamentous inclusions with β-sheet conformation in the core. Deposits can be fibrillar and insoluble (Chiti and Dobson, 2006), fibrillary but with some degree of solubility (Han et al., 2012), or amorphous. The composition of these deposits is often specific for each disease and composed of one predominant protein in each of these diseases, such as β-amyloid, tau, huntingtin, α-synuclein or prion protein (Goedert and Spillantini, 2006). These proteins will be further discussed in following subsections with relation to the disease the misfolded species are typically found in.

Degradation of misfolded proteins by the ubiquitin-proteasome system (UPS) or the autophagy-lysosome pathway (ALP; Labbadia and Morimoto, 2015; Yerbury et al., 2016) have been regarded as potential therapeutic targets for the treatment of neurodegenerative diseases, as aging leads to decreased efficiency of protein quality control (Ben-Zvi et al., 2009; Wang J. et al., 2009). Ubiquitination of misfolded proteins ultimately leads to degradation of proteins by the 26S proteasome and release of the ubiquitin chain (Pickart, 2001).

Proteins with longer half-life are predominantly degraded via the ALP. The role of chaperone-independent macroautophagy (which leads to the formation of autophagosomes) in the central nervous system of mammals is well-documented (Grant and Donaldson, 2009; He and Klionsky, 2009). In addition, chaperone-mediated autophagy (CMA) has been shown to protect against accumulation of tau, α-synuclein, and polyQ-huntingtin (Htt) in models of tauopathy, PD, and HD, respectively (Wang Y. et al., 2009; Qi et al., 2012; Xilouri et al., 2013). As shown by Agarraberes and Dice (2001), CMA requires the formation of complexes of chaperones and co-chaperones, including Hsp40, Hsp70, STI1/HOP, Hsp70-interacting protein (Hip), and Bcl2-associated athanogene 1 protein (BAG-1). Increasing evidence has shown that disrupted autophagy—either through autophagosomes or CMA—and lysosomal mechanisms contribute to the pathogenesis of AD, PD, HD, ALS, and FTD (Anglade et al., 1997; Nagata et al., 2004; Yu et al., 2005; Morimoto et al., 2007; Hu et al., 2010; Cuervo and Wong, 2014; Nah et al., 2015).

The following subsections will briefly discuss PD, HD, ALS, prion diseases and AD, and the effects of Hsp70, Hsp90, and STI1 (if investigated) on the misfolded protein species in each of these diseases and it is also summarized briefly in Table 1. We will also discuss how modulating the levels or activities of these chaperones affects protein toxicity and aggregative-capacity.

**Table 1:** **Overview comparing Hsp70, Hsp90, STI1, and/or Hsp40 protein quality control in various model organisms of neurodegenerative disease**.

**Disorder**	**Model**	**Hsp70**	**Hsp90**	**STI1**	**Hsp40**
Parkinson's disease	*In vitro* or cell line or yeast	↓α-synuclein fibril formation *in vitro* (Roodveldt et al., 2009). Heat shock-induced ↑ Hsp70, ↓α-synuclein inhibition of proteasome in human fibroblasts (Lindersson et al., 2004); Hsp70 overexpression in neuroglioma cells ↓α-synuclein oligomerization (Outeiro et al., 2008)	Hsp90 ↓α-synuclein fibril formation *in vitro* (Falsone et al., 2009); ↑ ROS in Hsp90 haploid deletion yeast mutants by α-synuclein (Liang et al., 2008); Hsp90 inhibition in neuroglioma ↓α-synuclein oligomerization and toxicity (Putcha et al., 2010)	STI1 ↓ monomeric A53T α-synuclein aggregation *in vitro* (Daturpalli et al., 2013)	
	Animal model	↑ Synuclein inclusions in Hip knockdown (Roodveldt et al., 2009), dependent on Hsp70			
		Hsp70 deletion in *D. melanogaster* ↑α-synuclein (Auluck et al., 2002); ↑ Hsp70 in mice ↓α-synuclein oligomerization (Klucken et al., 2004); Hsp70 injection into s.nigra ↓ dopaminergic cell loss in rats (Dong et al., 2005)			
Huntington's disease	*In vitro* or cell line or yeast	Hsp70 overexpression ↓ Htt aggregates and toxicity in yeast and various cell lines (Warrick et al., 1999; Carmichael et al., 2000; Jana et al., 2000; Krobitsch and Lindquist, 2000; Wacker et al., 2004)		STI1 overexpression in yeast ↓ Htt toxicity, promoted reorganization to foci, Hsp70/TPR1-dependent (Wolfe et al., 2013)	Hsp40 overexpression ↓ Htt aggregates and toxicity in yeast and various cell lines (Warrick et al., 1999; Carmichael et al., 2000; Jana et al., 2000; Krobitsch and Lindquist, 2000; Wacker et al., 2004)
	Animal model	Deletion of Hsp70 in mice ↑ inclusion body size (Wacker et al., 2009); Hsp70 overexpression ↓ Htt aggregates and toxicity; Hsp70 silencing in *C. elegans* ↑ Q35 aggregation (Brehme et al., 2014)	Hsp90 silencing in *C. elegans* ↑ Q35 aggregation (Brehme et al., 2014)	STI1 silencing in *C. elegans* ↑ Q35 aggregation (Brehme et al., 2014)	Hsp40 silencing in *C. elegans* ↑ Q35 aggregation (Brehme et al., 2014); Hsp40 expression in *D. melanogaster* ↓ Htt toxicity (Kazemi-Esfarjani and Benzer, 2000)
ALS	*In vitro* or cell line or yeast	Hsp70 binds and regulates TDP43 nuclear accumulation in HeLa (Freibaum et al., 2010; Udan-Johns et al., 2014); Hsp70 knockdown in N2a ↑ phospho-TDP43 and C-terminal fragment (Zhang et al., 2010); Heat shock-induced Hsp70 in HEK293 ↓ insoluble and hyperphosphorylated TDP-43 species (Chen et al., 2016)	Pharmacological Hsp90 inhibition in HeLa ↓ levels of full length TDP43 (Lotz et al., 2003)		Hsp40 binds and regulates TDP43 nuclear accumulation in HeLa (Freibaum et al., 2010; Udan-Johns et al., 2014); ↑ DnaJC5 in HEK293 ↑ disease-associated cleaved TDP43 secretion (Fontaine et al., 2016); Heat shock-induced Hsp40 in HEK293 ↓ insoluble and hyperphosphorylated TDP-43 species (Chen et al., 2016); ↓ Hsp40 in N2a ↑ phospho-TDP43 and C-terminal fragment (Zhang et al., 2010)
	Animal model	↓ Hsp70 levels in TDP-43 transgenic mouse line (Chen et al., 2016)			↓ Hsp40 levels in TDP-43 transgenic mouse line (Chen et al., 2016)
	Humans	↓ Hsp70 in sporadic cases *post-mortem* (Chen et al., 2016)			↓ Hsp40 in sporadic cases *post-mortem* (Chen et al., 2016)
Prion diseases	*In vitro* or cell line or yeast	↓ PS^+^ replication and propagation in yeast with mutation in Hsp70 allele (Jones et al., 2004)		STI1 deletion in yeast ↑ PS^+^ propagation (Jones et al., 2004; Reidy and Masison, 2010); STI1 reduces RNQ^+^ prion toxicity (Wolfe et al., 2013)	
	Animal model	↑ Astrocytic Hsp70 in scrapie injected mice (Diedrich et al., 1993); Overexpression of human Hsp70 in mice did not ameliorate prion pathology (Tamguney et al., 2008)			
	Humans	↑ Inducible Hsp70 in CJD, regions with less atrophy have ↓PrP^*Sc*^ and ↑Hsp70 (Kovacs et al., 2001)			
Alzheimer's disease	*In vitro* or cell line or yeast		*In vitro* Hsp90 competes with PrP for STI1 binding (Maciejewski et al., 2016); in primary murine neurons Hsp90 decreased STI1 neuroprotection against Aβ oligomers (Ostapchenko et al., 2013)	STI1 ↓ PrP^C^-AβO binding *in vitro* (Maciejewski et al., 2016); in HEK cells and primary neurons (Ostapchenko et al., 2013); STI1 protected primary murine neurons from AβO toxicity in PrP^C^/α7nAChRs-dependent way (Ostapchenko et al., 2013)	
	Animal model	Toxicity buffering against Aβ in *C. elegans* (Brehme et al., 2014)	Toxicity buffering against Aβ in *C. elegans* (Brehme et al., 2014); treating AD mice with 17-AAG improved synaptic marker density and memory Hsp90 inhibitors ↓ hyperphosphorylated tau (Chen et al., 2014; Wang et al., 2016)	Toxicity buffering against Aβ in *C. elegans* (Brehme et al., 2014); STI1 downregulation ↑ tau-induced neuron loss in *D. melanogaster* (Ambegaokar and Jackson, 2011); ↑ in hippocampus in APPswe/PS1dE9 mice (Ostapchenko et al., 2013)	Toxicity buffering against Aβ in *C. elegans* (Brehme et al., 2014)
	Humans			↑ In cortex *post-mortem* (Ostapchenko et al., 2013)	

### Chaperones in synucleinopathies

PD is the second most common neurodegenerative disease characterized mainly by loss of dopaminergic neurons that project from the Substantia nigra, although several other neuronal groups are also affected (Davie, 2008). Several genes have been linked to genetic forms of PD, and amongst them *SNCA*, that codes for α-synuclein is of particular interest. α-synuclein, a protein associated with neuronal membranes (Maroteaux and Scheller, 1991), can aggregate in plaques in AD, termed the non-amyloid component (Ueda et al., 1993). However, α-synuclein is more commonly associated with PD, where it forms filamentous intraneuronal inclusions composed of ubiquitinated and phosphorylated α-synuclein, a component of Lewy bodies (Trojanowski et al., 1998; Goedert, 1999). This results in loss of neurons, and of importance to symptoms in PD, dopaminergic neurons in the Substantia nigra are particularly affected (de Lau and Breteler, 2006; Dickson, 2012). Augmented levels of α-synuclein or α-synuclein-containing aggregates are also characteristic of other neurodegenerative diseases including Lewy body dementia, multiple system atrophy and AD (Halliday et al., 2011; Serrano-Pozo et al., 2011; Ingelsson, 2016), forming a group of diseases termed “synucleinopathies.” The involvement of molecular chaperones in PD was first suggested by the observation that Hsp90, Hsp70, Hsp60, Hsp40, and Hsp27 were localized in Lewy bodies (McLean et al., 2002; Uryu et al., 2006; Leverenz et al., 2007). Increasing evidence has shown protective actions of molecular chaperones against α-synuclein-induced toxicity both *in vitro* and *in vivo*. Lindersson et al. (2004) showed that filaments of α-synuclein are able to bind to a component of the proteasome (the 20S subunit) and selectively impede the chymotrypsin-like activities of the proteasome *in vitro*. Recombinant human Hsp70 was capable of binding these α-synuclein filaments and attenuating their chymotrypsin-like inhibitory activity. Furthermore, heat shock of fibroblasts expressing α-synuclein lead to a significant increase in Hsp70 levels, which also reduced the inhibitory effects of α-synuclein on the proteasome (Lindersson et al., 2004). Roodveldt et al. (2009) found that Hsp70 and Hsp70-interacting protein (Hip) prevented formation of α-synuclein fibrils *in vitro* and in *C. elegans* knockdown of Hip increased synuclein inclusions in an Hsp70-dependent manner. Interestingly, Hsp70 overexpression in human neuroglioma cells transfected with mutant α-synuclein led to 50% less oligomeric α-synuclein species (Outeiro et al., 2008). Transgenic expression of familial PD mutations (A30P and A53T in α-synuclein) in fruit flies causes a significant degeneration of dopaminergic neurons, but co-expression of human Hsp70 abrogated this loss (Auluck et al., 2002). Overexpression of Hsp70 in yeast and mouse models, and Hsp90 inhibition with geldanamycin in human cell lines has been shown to counteract formation and accumulation of α-synuclein oligomers and alleviate α-synuclein-induced toxicity (Klucken et al., 2004; McLean et al., 2004; Flower et al., 2005; Luk et al., 2008). Conversely, Auluck et al. (2002) also reported acceleration of α-synuclein toxicity after inducing a dominant negative mutation of fruit fly Hsp70, which further confirmed the critical role of Hsp70 in α-synuclein regulation. Similarly, injection of Hsp70 into the Substantia nigra of MPTP-treated rats (a toxin that results in similar pathologies to those seen in PD) prevented dopaminergic cell loss (Dong et al., 2005).

Much less is known about the role of Hsp90 in regulating α-synuclein aggregation. *In vitro* experiments show that Hsp90 can both abolish α-synuclein binding to vesicles and promote fibril formation in an ATP-dependent manner (Falsone et al., 2009). More recent i*n vitro* experiments investigating Hsp90 interaction with the A53T mutant of α-synuclein revealed that all three Hsp90 domains bind to and prevent A53T α-synuclein aggregation. However, Hsp90 could not bind to monomeric or fibrillary synuclein species in this model (Daturpalli et al., 2013). Interestingly, haploid deletion of yeast Hsp90 (Hsp82) enhanced α-synuclein toxicity, specifically, by increasing reactive oxygen species accumulation (Liang et al., 2008). However, Putcha et al. (2010) showed that Hsp90 inhibition with 17-AAG (a geldanamycin derivative), which leads to upregulation of Hsp70, prevented α-synuclein oligomer formation and toxicity in the H4 neuroglioma cell line. Due to STI1 ability to modulate Hsp70/Hsp90 activity, Daturpalli et al. (2013) conducted an *in vitro* experiment to assess if STI1 could have its own effect on α-synuclein aggregation. STI1 could only attenuate monomeric A53T α-synuclein aggregation *in vitro* (Daturpalli et al., 2013). This suggests that STI1 is capable of having some of its own chaperone-like activity, but interaction with Hsp70 or Hsp90 would have a greater effect on reorganization of toxic α-synuclein species. The literature thus far suggests that increasing Hsp70 levels by activating the heat shock response or by genetic manipulation would be a suitable method for reducing α-synuclein toxicity. This could prove beneficial in reducing toxicity-related symptoms.

### Chaperones in huntington's disease

Excess CAG repeats in the IT15 gene is a heritable mutation that causes HD and leads to the accumulation of huntingtin protein [Huntingtons's Disease Collaborative Research Group (1993)]. Protein deposits of mutated huntingtin form inclusion bodies within motor neurons in the spinal cord, as well as neurons in the cerebellum, cortex and striatum (Davies et al., 1997). Recent work suggests that the sequestering of toxic huntingin (Htt) into inclusion bodies may be a way to remove this toxic species as increased formation correlated with a reduction in levels of toxicity and neuronal death (Arrasate et al., 2004; Miller et al., 2010).

HD is part of the polyQ group of neurodegenerative diseases, which includes spinocerebellar ataxias, spinal and bulbar muscular atrophy (also known as Kennedy's disease), and dentatorubral-pallidoluysian atrophy (Williams and Paulson, 2008). In a fly model, Kazemi-Esfarjani and Benzer (2000) showed transgenic vector-mediated suppression of Htt toxicity by the molecular chaperones dHDJ1, a homolog of human Hsp40, and dTPR2, a homolog of human tetratricopeptide repeat protein 2. Likewise, deletion of Hsp70 in mice increased the size of polyQ inclusion bodies (Wacker et al., 2009). In addition, overexpression of Hsp40 and/or Hsp70 suppressed polyQ-dependent aggregation and neurodegeneration in cell cultures, yeast, fly, and mouse models (Warrick et al., 1999; Carmichael et al., 2000; Jana et al., 2000; Krobitsch and Lindquist, 2000; Wacker et al., 2004). STI1 overexpression in yeast suppressed Htt toxicity and drove the re-organization of small Htt103Q foci into larger assemblies through interaction with Hsp70, whereas STI1 deletion aggravated Htt toxicity and hampered foci formation (Wolfe et al., 2013). Specifically, point mutations (A31T or G76N) in the TPR1 domain of yeast STI1 (Hsp70 interacting domain) prevented reorganization of Htt to STI1 foci, which resulted in a significant reduction in cell growth. This indicates that functional STI1 interaction with Hsp70 is required for Htt103Q reorganization and toxicity buffering. In *C. elegans* expressing the Q35 aggregate prone-protein, siRNA for Hsp40, Hsp70, Hsp90, or STI1 significantly increased the number of Htt aggregates (Brehme et al., 2014). Therefore, there is strong evidence for an important role of chaperones and co-chaperones as therapeutic targets in HD.

### Chaperones in amyotrophic lateral sclerosis

ALS is a fatal neurodegenerative disorder that affects motor neurons of the brainstem, cortex and spinal cord, and results in weakness and atrophy of voluntary skeletal muscles (Paez-Colasante et al., 2015). ALS can be divided into two major classes—either the disease presents sporadically, which is 90% of cases, or it can be inherited. There are a number of proteins, RNAs and miRNAs dysregulated in ALS. The first aggregated protein to be identified was Cu/Zn superoxide dismutase (SOD1; Rosen et al., 1993), then trans-active DNA binding protein-43 (TDP-43; Arai et al., 2006; Neumann et al., 2006), along with fused in sarcoma/translocated in liposarcoma (FUS; Kwiatkowski et al., 2009; Vance et al., 2009), see Blokhuis et al. (2013) for a more extensive review on toxic protein accumulation in ALS.

However, in most familial or sporadic cases of ALS, the RNA binding protein TDP-43 shows signs of mislocalization and aggregation. TDP-43 is capable of binding to DNA and RNA, making it a key regulator of transcription, translation and cellular growth (Ayala et al., 2011; Polymenidou et al., 2011). TDP-43 can mislocalize to the cytoplasm, be ubiquitinated, hyperphosphorylated and ultimately form aggregates (Neumann et al., 2006; Mackenzie et al., 2007; Sreedharan et al., 2008; Brettschneider et al., 2013). Mutations in TDP-43 have been linked to ALS and FTD (Arai et al., 2006; Neumann et al., 2006). All together these results suggest the need to better understand the relationship between TDP-43 and chaperones.

TDP-43 carries out most of its functions in the nucleus, but it can be transported to the cytosol due to the nuclear export sequence near its N-terminus (Ayala et al., 2008). TDP-43 contains two RNA recognition motifs in the core of the protein and a C-terminal domain that contains a glutamine/asparagine-rich prion-like region that cooperates in protein-protein interactions (Ou et al., 1995; Budini et al., 2012a,b; Mackness et al., 2014). This prion-like domain allows for association with other TDP-43 molecules (Budini et al., 2012b) and is becoming a major area of research in neurodegenerative diseases involving TDP-43 associated pathology. Deletion of the prion-like domain of TDP-43 in HeLa cells eliminated heat shock induced nuclear aggregation, and further deletion of a glycine-rich region in this domain significantly reduced cytosolic mislocalization of the toxic 25 kDa TDP-43 variant (Udan-Johns et al., 2014). Recent studies in HeLa cells showed that Hsp40 and Hsp70 constitutively bind to and regulate the nuclear aggregation of TDP-43 (Freibaum et al., 2010; Udan-Johns et al., 2014).

Using HEK293T cells Fontaine et al. (2016) investigated the roles of the constitutively expressed Hsp70 homolog Hsc70 and its co-chaperone DnaJC5 in the secretion of neurodegenerative-disease associated proteins. Secretion of these proteins by unconventional mechanisms is thought to contribute to their spreading in the brain. DnaJC5 supports secretion via the Soluble NSF Attachment Protein Receptor (SNARE) complex at synapses in a calcium-dependent manner (Jacobsson and Meister, 1996; Chamberlain and Burgoyne, 1997, 1998; Umbach and Gundersen, 1997; Weng et al., 2009; Sharma et al., 2012). Overexpression of DnaJC5 lead to significant secretion of WT and disease associated mutants of TDP-43 from HEK cells (Fontaine et al., 2016) and this was dependent upon functional Hsc70. Interference with this mechanism could potentially regulate the spreading of misfolded TDP-43 in the brain. However, to date there has been limited experiments in neuronal cells, neurons or in animal models to test these findings obtained in non-neuronal cells.

Both Hsp70 and Hsp90 can be co-immunoprecipitated with TDP-43. Moreover, knockdown of Hsp70 or Hsp90 in human neuroblastoma cells lead to a significant increase in C-terminal and phosphorylated TDP-43, which are toxic TDP-43 species known to aggregate in the cytoplasm (Zhang et al., 2010). Treating HeLa cells with celastrol, an Hsp90 inhibitor, reduced levels of full length TDP-43, specifically by impairing Cdc37 (an Hsp90 co-chaperone which aids in client docking; Lotz et al., 2003)—Hsp90 interaction with TDP-43 (Jinwal et al., 2012). Recent work by Chen et al. (2016) further supported the role of Hsps in TDP-43 regulation, whereby activation of the heat shock response, by overexpression of HSF1 in HEK cells increased levels of Hsp70 and Hsp40, which lead to increased clearance of insoluble and hyperphosphorylated TDP-43. Interestingly, TDP-43 misregulation has also been found in a proportion of patients with AD (Amador-Ortiz et al., 2007; Wilson et al., 2011). hnRNP A2/B1 and A1, which are RNA-binding proteins that interact with TDP-43, have been shown to be decreased in AD, due to abnormal regulation of cholinergic signaling (Berson et al., 2012; Kolisnyk et al., 2013, 2016a,b). Although we have started to understand how chaperones and co-chaperones may regulate TDP-43, their role in neurons and other brain cells has not yet been examined in detail.

A significant reduction in Hsp70 and Hsp40 protein levels is observed in the brains of TDP-43Q331K transgenic mouse model of ALS and patients with sporadic ALS (Chen et al., 2016). HSF1 protein levels were also reduced in mice, but not in human brains (Chen et al., 2016). This suggests that, in disease, the heat shock response may be compromised and thus contribute to the accumulation of insoluble TDP-43 protein aggregates.

### Chaperones and prions

In prion diseases, PrP^C^ is converted into PrP^*Sc*^, which can work via template-mediated misfolding to further convert host PrP^C^ protein into a variety of misfolded forms that aggregate and accumulate within the nervous tissue (Will and Ironside, 1999; Budka, 2003; Soto and Castilla, 2004; Linden et al., 2008). Misfolding of PrP^C^ results in a class of diseases called transmissible spongiform encephalopathies (TSEs). Prion disease can arise sporadically, from genetic mutation or through transmission, such as by consumption of prion-infected tissues. TSEs include bovine spongiform encephalopathy in cattle, as well as sheep scrapie and variant CJD in humans (Linden et al., 2008). PrP^C^ contains a disordered N-terminal domain and a globular C-terminal domain that is largely α-helical (Riek et al., 1996, 1997).

In order to better understand prion propagation and chaperone regulation in a simple eukaryotic model, yeast prions have been extensively studied. Yeast prions are self-propagating amyloid forms of soluble proteins that can function as protein-based inheritable elements. The yeast prion, PSI^+^, is a transmissible, self-replicating, and aggregation prone mutant of yeast translation termination factor Sup35p (Glover et al., 1997; King et al., 1997). Jones et al. (2004) found that a mutation in the SSA1 Hsp70 allele (SSA1–21p) significantly impaired PSI^+^ self-replication and propagation. Interestingly, in this cell line, deletion of yeast STI1 regenerated PSI^+^ propagation. Conversely, overexpression of STI1 reduced the mitotic capacity of PSI^+^ prions (Jones et al., 2004). Additionally, overexpression of Hsp104 is capable of eliminating PSI^+^ prions (Chernoff et al., 1995), but this is dependent upon expression of STI1 (Reidy and Masison, 2010). Deleting STI1 had no effect on levels of Hsp104, but eliminated Hsp104 “curing” activity (Reidy and Masison, 2010). Specifically, STI1 coordination of Hsp70 and Hsp90 was responsible for this prion elimination activity, as mutations in the TPR1 and TPR2 domains of STI1 lead to a drastic increase in PSI^+^ propagation. This suggests that STI1 coordination of Hsp70-Hsp90 as well as Hsp104 activity is required for disaggregation of yeast prions. Furthermore, STI1 expression and activity was also found to reduce toxicity of Rnq1 (a yeast protein with a glutamine-rich prion domain) prions, RNQ^+^ (Wolfe et al., 2013). STI1 recruited RNQ^+^ prions to foci containing Hsp104, amyloid like proteins and Hsp40, ultimately buffering toxicity by these prions.

As with many of the misfolded proteins that cause neurodegenerative diseases, knowledge of the physiological functions of PrP^C^ is still not complete. Knockout of PrP^C^ in mice affects synaptic transmission (Maglio et al., 2006), causes gross demyelination in the sciatic nerve specifically due to PrP^C^ depletion in neurons (Bremer et al., 2010), and alterations in sleep pattern (Tobler et al., 1996). Elimination of PrP^C^ also protects mice against infection with PrP^Sc^ (Bueler et al., 1993).

C57BL6 mice injected with 22L strain of scrapie had a significant increase in protein levels of inducible Hsp70 in active astrocytes (Diedrich et al., 1993). Similarly, mice infected with forms of scrapie known to induce plaques and increased vacuolation, had a significant increase in Hsp70 RNA expression toward the terminal phases of infection (Kenward et al., 1994). Kovacs et al. (2001) found increased immunoreactivity of inducible Hsp70 in Purkinje cells from CJD patients, and regions with higher levels of Hsp70 had less spongiform-like atrophy and increased levels of PrP^C^ rather than PrP^Sc^. This suggests a potential neuroprotective effect of Hsp70.

Tamguney et al. (2008) conducted a seminal study on 20 potential gene candidates that could regulate the replication of prions in mice infected with scrapie or cow 301V prions. Genes were selected based upon known interactions with PrP^C^ in a diseased or non-diseased state, significant upregulation in prion disease, post-translational modification of PrP, or involvement in PrP^C^-related signal transduction (Tamguney et al., 2008). Interestingly, overexpression of human Hsp70 had no effect on prion disease onset.

To our knowledge there have been almost no studies investigating the role of Hsp90 and its co-chaperones in prion diseases. STI1 can signal via the prion protein as discussed above, and prion infection in cells abolishes STI1 signaling via the prion protein (Roffe et al., 2010). Interestingly, interaction of Hsp90 with STI1 also decreases PrP^C^-dependent STI1 neuroprotection (Maciejewski et al., 2016), which suggests that secreted Hsp90 may interfere with STI1 interaction with PrP^C^. Given that STI1 regulates protein aggregates via its co-chaperone activity (Wolfe et al., 2013), and also has extracellular cytokine-like neurotrophic function, it is likely that its effects on prion diseases and other neurodegenerative diseases are complex. By further understanding the cause and mechanism of the aggregation of these proteins, interventions targeting the chaperone machinery toward refolding or degradation could be utilized.

### Chaperones in AD

AD is the most common form of dementia, particularly affecting the aging population. Pathologically, it is defined by accumulation of two types of protein aggregates in the forebrain; extracellular plaques of Aβ, and intraneuronal neurofibrillary tangles (NFT) of microtubule-associated protein tau (Selkoe, 1991). As is the case with other diseases associated with misfolded proteins, analysis of AD brains and AD animal models revealed increased levels of Hsps and their co-chaperones, including Hsp27 (Renkawek et al., 1994), Hsp70 (Perez et al., 1991), and STI1 (Ostapchenko et al., 2013). A significant effort was made by a number of researchers to test whether chaperone system participates, directly or indirectly, in the pathogenic processes of Aβ and tau misfolding.

#### Aβ peptide generation and toxicity

Aβ peptides consisting of around 39–43 residues are formed by proteolytic cleavage of its precursor, APP, by beta-site APP cleaving enzyme (BACE, β-secretase) and by a γ-secretase complex, formed by several proteins including presenilins (O'Brien and Wong, 2011). Aβ peptide toxicity was originally thought to be related mainly to the amyloid plaques that form throughout cortex and hippocampus in AD. However, during the last two decades it has also become recognized that soluble Aβ oligomers (AβOs) are toxic to synapses (Lambert et al., 1998; Walsh et al., 2002; Ferreira and Klein, 2011; Benilova et al., 2012; Mucke and Selkoe, 2012; Lesne et al., 2013). Aβ oligomers are thought to increase early before plaque formation and correlate with the onset of the neurotoxic events, such as excitotoxicity, synaptic loss as well as impairment of LTP and learning/memory in rodent models (Lambert et al., 1998; Klein et al., 2001; Klein, 2002; Walsh et al., 2002; Wang et al., 2005). Synaptotoxicity by AβOs depends on their interaction and corruption of multiple neuronal receptors, an effect that seems to depend on the initial interaction with PrP^C^ (Lauren et al., 2009; Gimbel et al., 2010; Caetano et al., 2011; Kudo et al., 2012; Um et al., 2012; Ostapchenko et al., 2013; Beraldo et al., 2016). AβO/PrP^C^ can engage metabotropic glutamate receptor 5 (Um et al., 2012, 2013; Beraldo et al., 2016) to activate pathogenic intracellular pathway that leads to activation of Fyn kinase, NMDA receptor mistrafficking, excitotoxicity and LTP inhibition.

Significant effort by several research groups were aimed to prevent AβO toxicity, employing anti-Aβ_42_/AβO (reviewed in Wisniewski and Drummond, 2016) and anti-PrP^C^ (Chung et al., 2010; Barry et al., 2011) antibodies and N-terminal fragment of PrP^C^ (Beland et al., 2014). However, to our knowledge, none of these potential therapies passed or even reached clinical trials yet. Remarkably, extracellular STI1, can bind to PrP^C^ and activate α7 nicotinic acetylcholine receptor (nAChR), which mediate STI1-PrP^C^ neurotrophic effects, efficiently preventing the binding of AβOs to PrP^C^ on the neuronal surface, as well as general binding of these oligomers to neurons (Ostapchenko et al., 2013). Due to this effect, as well as protective signaling via α7 nAChR/PrP^C^ complex, STI1 completely blocks AβO/PrP^C^ toxicity *in vitro* (Ostapchenko et al., 2013). Whether Hsp70/Hsp90/STI1 exist extracellularly in AD brain separately or as a complex is unknown, but one may expect complex effects of extracellular chaperones on Aβ aggregation and toxicity in AD brain. To start with, we found in a biochemical assay that Hsp90 modulates formation of the STI1/PrP^C^ complex, possibly resulting in decreased STI1 neurotrophic signals (Maciejewski et al., 2016).

Many *in vivo* AD models, including those that employ invertebrate and mammal species, are based on Aβ toxicity. In *C. elegans* Aβ expression leads to formation of peptide deposits and decreased motility (Link, 1995). This toxicity can be rescued by blocking the insulin growth factor-like signaling pathway, with a major role being played by HSF1 (Cohen et al., 2006). In this study, Cohen and colleagues showed that treatment with HSF1 RNAi increased Aβ toxicity in worms, probably due to increased amount of neurotoxic Aβ aggregates. The question remains, which mechanism activated by HSF1, plays a role in increased Aβ toxicity. Morimoto and colleagues approached this question by analyzing the various chaperones in worms expressing Aβ (Brehme et al., 2014). Systematic knockdown of Hsps and co-chaperones showed that Hsp40, Hsc70, Hsp90, and STI1, while not affecting motility in young animals, seem to normally buffer Aβ toxicity in *C. elegans*, as well as to alleviate age-related decrease in worm motility. Of interest, these chaperones and co-chaperones form an expression network in human brains, but the connecting links are significantly weakened in both AD and normal aging (Brehme et al., 2014), suggesting dysfunctional chaperone activity with age and disease.

The role of HSF1 in AD suggested by the results in *C. elegans* was recently supported by work with AD mouse models studying the effect of Hsp90 inhibitors on Aβ synaptotoxicity and behavioral impairment. Treatment of AD mice with 17-AAG (Chen et al., 2014) or OS47720 (Wang et al., 2016), Hsp90 inhibitors improved synaptic markers and density, *in vivo* LTP and memory loss and these effects were mediated by HSF1 activation and upregulation of synaptic genes. In contrast to the effect of HSF1, it is remarkable that whereas in *C. elegans* knockdown of Hsp90 is deleterious, in mammals inhibition of Hsp90 can actually improve Aβ-mediated toxicity.

Interestingly HSF1, which under stress conditions induces expression of Hsp70, Hsp90 and other chaperones, also upregulates production of APP (Dewji and Do, 1996). HSF1, besides its role in upregulation of heat shock machinery, is known as a major factor facilitating synaptic fidelity (Hooper et al., 2016). It is possible that HSF1-induced APP upregulation may be due to the pro-synaptogenic activity of this transcription factor. Indeed, APP has been shown to serve as a cell adhesion molecule (Small et al., 1999). On the other hand, synaptic activity by itself affects APP trafficking, routing it toward synapses (Tampellini et al., 2009). Besides, the same study showed that in neurons overexpressing APP with the Swedish familial mutation, synaptic activity also decreases intraneuronal Aβ. This result is paralleled by findings that activation or de-activation of synaptic activity, increases or decreases Aβ secretion, respectively (Kamenetz et al., 2003; Cirrito et al., 2005; Bero et al., 2011; Li et al., 2013; Yuan and Grutzendler, 2016). Considering the deleterious effect of Aβ on synaptic activity and integrity, HSF1, APP, and Aβ may form a self-regulating mechanism for controlling neuronal function.

#### Tau

Significant influence of Hsp70/90 machinery on AD pathology is implemented via microtubule associated protein tau. Physiologically, tau acts as a major regulator of microtubule formation (Weingarten et al., 1975) and in the CNS, tau is typically found in the cytoplasm or axons (Binder et al., 1985), where it promotes outgrowth and stabilizes microtubule formation. Tau is abnormally phosphorylated in AD due to increased activity of GSK-3 and other tau kinases (Alvarez et al., 1999; Avila et al., 2010; Tremblay et al., 2010; Cavallini et al., 2013), likely as a result of initial Aβ toxicity (Tamagno et al., 2003; Ryan et al., 2009; Hernandez et al., 2010). Hyperphosphorylated tau forms paired helical filaments, which are the main component of neurofibrillary tangles (Grundke-Iqbal et al., 1986a,b; Cao and Konsolaki, 2011), a critical pathological hallmark in AD. A number of studies have shown that these hyperphosphorylated tau species can be recognized by Hsps and their co-chaperones, including Hsp27, Hsp70, CHIP, and αB crystalline, in order to repair malignant tau or proceed with its recycling (Dou et al., 2003; Dabir et al., 2004; Petrucelli et al., 2004; Shimura et al., 2004; Luo et al., 2007). Recently, the structure of Hsp90-tau complex has been resolved (Karagoz et al., 2014). It explained how Hsp70 and Hsp90 can simultaneously bind to the intrinsically unstructured tau, making use of the atypically large substrate-binding site on Hsp90, which is rather open and accessible to clients such as tau. Of interest, low affinity hydrophobic connections in the Hsp90 substrate binding site could explain a general principle of Hsp90 interaction with disordered substrates or folded proteins.

Interestingly, inhibitors of Hsp90 decrease levels of phosphorylated tau, suggesting that Hsp90 may protect hyperphosphorylated tau from degradation (Dickey et al., 2006). Inhibition of Hsp90 in HeLa cells transfected with mutant tau (P301L) increased CHIP complex formation with phosphorylated tau (p-tau) and CHIP selectively degraded these p-tau species, essentially preventing aggregation of p-tau (Dickey et al., 2007). CHIP is also highly colocalized with p-tau and neurofibrillary tangles (aggregates of hyperphosphorylated tau; Dickey et al., 2007). These findings make CHIP a suitable candidate for modulating tau activity in neurodegenerative tauopathies, especially due to its ubiquitin enzyme activity. On the other hand, a complex of Hsp90 with the co-chaperone FKBP51 protected tau from proteasomal degradation and correlated with the neurotoxic tau species (Jinwal et al., 2010; Blair et al., 2013). FKBP51 overexpression decreased the amount of tau tangles in P301L tau transgenic mice, but increased soluble tau, including oligomeric and hyperphosphorylated species. This in turn led to increased tau toxicity, reflected in neuronal loss in P301L mice hippocampus and in decreased proliferation of tau-expressing neuronal cultures (Blair et al., 2013). Dickey and colleagues also found that FKBP51 expression is increased with age and in AD (Blair et al., 2013). This led them to hypothesize that Hsp90 interaction with FKBP51 is altered in aging and AD brains, allowing for the preservation of soluble, but possibly neurotoxic protein species. Another member of FKBP family, FKBP52, may also be involved in tau-related neurodegeneration. Recent evidence suggests that FKBP52 is a key regulator of tau association with microtubules, specifically in inhibiting this function (Chambraud et al., 2010). Moreover, a significant reduction in tau-mediated neurite outgrowth was observed in cells overexpressing FKBP52 (Chambraud et al., 2010).

Alternatively, Hsp70 promotes tau stability and association with microtubules at high levels of expression (Dou et al., 2003; Jinwal et al., 2009). STI1 may also be important for protection against aberrant tau species, as its downregulation in fruit flies worsened tau-induced retinal degeneration (Ambegaokar and Jackson, 2011). Upregulation of both Hsp70 and Hsp90 increases tau association with microtubules (Dou et al., 2003). Of note, this study used geldanamycin-induced Hsp90 inhibition, which resulted in increased Hsp70/90 expression due to HSF1 activation. As HSF1 activates multiple members of Hsp machinery, it is difficult to draw conclusions as to which particular chaperone affected tau-microtubule coupling. Soluble levels of tau correlate with those of Hsps and their co-chaperones, while in tauopathies where total levels of tau increase, Hsp70/90 decrease (Dou et al., 2003). Overall, tau regulation by the Hsp machinery is very complex and careful analysis of all possible effects on tau is needed when considering an anti-AD therapy that modulates this machinery.

## Conclusion

In summary, the common observation of misfolded and aggregated proteins in neurodegenerative disease suggests dysregulation of chaperone activity. The balance between levels of Hsp70 and Hsp90 are becoming a major area of investigation, as both upregulation of Hsp70 and inhibition of Hsp90 in mammals reduce protein aggregation and toxicity. STI1 should be further investigated in models of protein aggregation, as STI1-PrP^C^ interaction results in neuroprotection, attenuates AβO toxicity, and STI1 is an irreplaceable co-chaperone for the Hsp70/Hsp90 machinery. Ultimately, much is still unknown about how to effectively control protein misfolding and prevent aggregation by targeting chaperones and co-chaperones in neurodegenerative disease. Further, investigation of chaperones and their partners using new mouse models, could help to elucidate the underlying mechanisms of these proteinopathies and allow for generation of effective and unambiguous pharmacological therapies.

## Author contributions

RL, AM, JML, and VO literature review, wrote the manuscript; WC, MD, and VP edited the manuscript; MP Literature review, wrote, and provide editing and scientific direction for the manuscript.

## Funding

Work supported by Canadian Institute of Health Research (MOP 93651, MOP 136930, MOP 126000, MOP 89919), NSERC (402524-2013), Brain Canada, Weston Brain Institute—Canada, Canadian Foundation for Innovation, and Ontario Research Fund (MP and VP). RL and AM received support from OGS.

### Conflict of interest statement

MP is an Associated Editor for the Frontiers Section on Neurodegeneration. The other authors declare that the research was conducted in the absence of any commercial or financial relationships that could be construed as a potential conflict of interest.
